# When a physicist wanders into biology…: an interview with KC Huang

**DOI:** 10.1186/s12915-018-0595-3

**Published:** 2018-11-01

**Authors:** Kerwyn Casey Huang

**Affiliations:** 1 Bioengineering Department, Stanford University, Stanford, CA USA; 2 Department of Microbiology and Immunology, Stanford University School of Medicine, Stanford, CA USA; 3 Chan Zuckerberg Biohub, San Francisco, CA USA

**Keywords:** Bacteria, Cell shape, Mentoring, Microbiome, Biophysics, High-throughput biology

## Abstract

Kerwyn Casey (“KC”) Huang is an Associate Professor at Stanford University, studying the physical nature of biological systems and the underpinnings of fundamental processes such as cell shape determination, cell division, and intracellular and microbial community organization. In this interview, KC discusses how the ability to pursue insights at scales from molecules to cellular communities can shed new light on longstanding questions, the necessity for new tools in exploring the microbiome, how to create an empowering lab environment, and why integrating chemistry with physics and biology can bring us closer to asking the right questions.

## What are the questions driving your research?

I have always been fascinated by simple, fundamental questions about how nature works. I focused on purely physical questions at first (how does a surface melt at the atomic scale?), but as a postdoc I accidentally wandered into biology and became hooked on a new set of unknowns. Many of the basic questions that my lab and I tackle now have been around for decades (if not centuries): how do cells break symmetry, how do they deal with starvation, how do they adjust to changes in temperature, pH, and osmolarity? It’s exciting to examine these biological questions with a multitude of new tools that allow us to explore scales all the way from single amino acids to communities of cells. If you had told me 10 years ago that I would be examining crystal structures in the morning and mouse tissue sections in the afternoon, I’d have said you were crazy. Now I can see that many seemingly different biological phenomena are actually related across space and time.

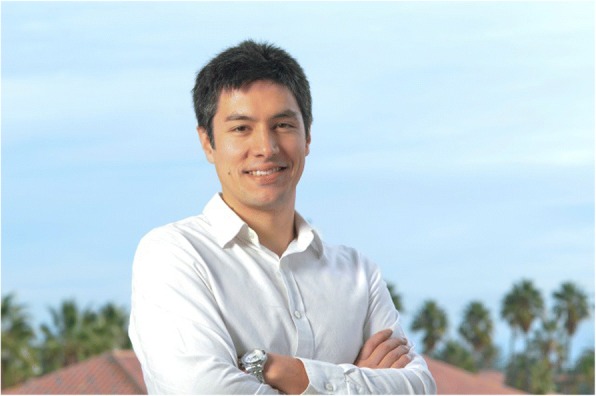



**Fig. 1.** KC Huang. Website: http://whatislife.stanford.edu/

## Looking back, is there a project that your lab pursued that stands out for you as particularly inspiring, tough, or simply memorable?

The clichéd (but true) answer is that every project has provided inspiration, tough challenges, and memories—in part because so many of our projects ended up taking us places we could never have predicted. However, two projects that laid the foundation for our lab today come to mind.

The first project was initialized by my former postdoc Rico Rojas (now an Assistant Professor at NYU), who was inspired by work in plants that suggested that turgor pressure (osmotic swelling) drives growth. Rico set out to determine how changes in turgor pressure affect the growth of bacteria. Almost a hundred years ago, Heinrich Walter discovered that bacterial colonies grow more slowly on agar plates at higher osmolarity, and one interpretation of this result is that the slowdown is due to the cells having lower turgor pressure. However, there are many other explanations involving transcriptional or structural adaptations. Rico used a microfluidic device to lower turgor pressure to zero almost instantaneously. To our surprise, not only did *Escherchia coli* cells not stop growing, they didn’t slow down at all [1]. I learned the simple lesson that the best way to interrogate how and why a cell cares about some environmental parameter is to change the environment very quickly and watch how the cell responds. I also gained an important appreciation for how new tools empower us to look at old data in new ways.

The second project emerged from conversations between myself and close friend and collaborator Justin Sonnenburg (Associate Professor of Microbiology & Immunology here at Stanford) as we prepared to teach a class on computational modeling of microbial communities. This class was as much an opportunity for the two of us to learn about each other’s expertise as it was to teach the students (this is the secret about great teaching: the instructors learn a lot too!). In talking with Justin about the gut microbiota, my mind naturally gravitated toward questions about the physical organization and dynamics of this organ. I was shocked by how little was known about these questions. This knowledge gap spurred our first collaboration, in which we developed computational and experimental tools to quantify the spatial organization of the mammalian gut. I was thrilled when our Teaching Assistants and the students in the course made important contributions to that project—again, this is the way that good teaching works. We made some really fun discoveries, such as the dramatic effects of dietary changes on the host mucosal lining and bacterial clustering [2]. Moreover, this experience unlocked a cornucopia of questions about the fundamental principles that guide the assembly, resilience, and evolution of the microbiota and how it responds to perturbations [3].

## Is there a paper or a scientist that inspired you, or was seminal for your research?

I was trained as a physicist, so I encountered every paper that I read as I first started thinking about biology with a sense of wonder. This transition required me to reinvent myself as a scientist—a process that is ongoing even today. As a result, I especially admire and am inspired by other scientists who have undergone a renaissance. My postdoc advisor Ned Wingreen (Princeton University) made seminal contributions to condensed matter physics, and then transitioned to biophysics as a research scientist at NEC Research Laboratories. Ned has worked at the frontiers of a diverse range of fields including chemotaxis, self-organization, quorum sensing, liquid–liquid separations, and many others. Ned commits to identifying and answering the most important questions in any field, regardless of whether they are trendy at the time. I’ve sought to work this way myself, and to inspire my lab members to have the same intellectual courage.

In the last several years, I’ve had the privilege of working with Carol Gross at UCSF. Carol has had an amazing career studying fundamental cellular components such as the heat shock response and RNA polymerase. And yet, around 10 years ago she completely shifted her lab to focus on the systems biology of bacterial stress responses. One of her first papers in this area is called the “Phenotypic Landscape of a Bacterial Cell” [4] and it’s such a tour-de-force that we literally look up data from that paper on a weekly basis. I hope that over the course of my career, I’m able to experience the excitement of such scientific shifts on a regular basis.

Last but certainly not least, I’m inspired by all the students and postdocs in my lab who take on the scientific challenges that we pursue. Their willingness to embrace the great unknown—and to invent the ways to navigate it—is what has made our lab successful.

## What are your guiding principles for running a lab? Do you have any advice to share with our readers?

Establishing a healthy work environment goes a long way toward having a happy, productive lab. For me, this means that all my lab members feel empowered to speak up when they have questions. We balance their scientific independence with a regular pattern of dialog between us. Above all, it’s important to me that my lab members are excited about their projects. Nearly all of my lab members are pursuing and publishing on multiple projects, and many of them collaborate closely with other labs as well. As a result, they are exposed to different ways of thinking about problems and a broad range of skills and tools, and they start forming their scientific network early on. In other words, I view my lab as a safe environment that encourages excellence in the skills that empower people to go on to be pioneers, deep thinkers, and good colleagues. My lab members frequently ask me how I deal with so many meetings each day, and I tell them that all those opportunities to discuss science is why I do this job.

## If you could, what would you tell your younger self?

Learn chemistry. The more we think about how cells interact with their environment, the more I think about the omnipresent and ever-changing chemical milieu that envelops the cell. Someone recently told me that there are two types of scientists: people who study metabolism, and people who don’t know that they’re studying metabolism. I’m glad we’re finally in the first group, but I still have a long way to go to actually have intuition about how chemistry affects biology. My wife took a Biology major and a Chemistry minor in college, and when we were first dating, I saw a silver necklace on Etsy that was shaped as the chemical structure of a polypeptide. I showed it to her, and she read off the word “GEEK” as if it was written in English. I aspire to be that geeky. When you have a strong intuition about the way the universe works, you are more quickly attracted to the questions that really matter.

## What are the most urgent questions you’d like to see addressed in your field?

My lab straddles many fields, but here I’ll answer for the microbiome field. Although microbiome research is built around cutting-edge -omics tools such as sequencing and mass spectrometry, there are so many areas in which new tool development would have a transformative impact. For example, there are tens of thousands of genes with novel function across the gut commensal bacteria, and yet our ability to perform genetic manipulation is currently limited to a few model organisms. Basic questions, such as which cells are growing when and where, remain unanswered because we lack tools to probe the gut spatially and dynamically. And even though sequencing can provide an amazing amount of insight, it is frustrating to have to wait for weeks or even months to examine data characterizing the gut community. With a turnaround of hours, long (multi-week) experiments could actually be adjusted on the fly.

## Are there any innovations in publishing you’d like to see happen?

In the best cases, I believe that scientific publishing makes for better science: critical but fair reviewers help to improve the quality of the paper, and publication is viewed as an accomplishment to be celebrated by the authors and field (regardless of the journal). Having said that, most of us have worst-case experiences that are often independent of the science itself: the third reviewer having a bad day, or the “what to do” moment when another just-published paper has significant overlap with a paper you are about to send out for review. Some journals are now encouraging communication among reviewers, which helps to highlight consensus criticism rather than the outlier concerns. Some journals are adopting a policy in which almost-concurrent papers on the same topic are viewed as a welcome reproduction of science rather than a pre-empting of novelty. I’d like to see more of these sorts of community-themed innovations (I think of them more as “improvements”), which seem to arise from constructive dialog among editors, reviewers, and authors.

**Website:** http://whatislife.stanford.edu/

## References

[1] Rojas E, Theriot JA, Huang KC (2014). Response of *Escherichia coli* growth rate to osmotic shock. Proc Natl Acad Sci U S A.

[2] Earle KA, Billings G, Sigal M, Lichtman JS, Hansson GC, Elias JE (2015). Quantitative imaging of gut microbiota spatial organization. Cell Host Microbe.

[3] Tropini C, Moss EL, Merrill BM, Ng KM, Higginbottom SM, Casavant EP (2018). Transient osmotic perturbation causes long-term alteration to the gut microbiota. Cell.

[4] Nichols RJ, Sen S, Choo YJ, Beltrao P, Zietek M, Chaba R (2011). Phenotypic landscape of a bacterial cell. Cell.

